# Case Report: The clear cell variant of papillary thyroid carcinoma: a clinicopathologic study of four cases with emphasis on *RET* gene fusions

**DOI:** 10.3389/fonc.2026.1787485

**Published:** 2026-04-23

**Authors:** Li Xu, Bin Luo, Qianwen Wang, Wen Liu, Xiaokang Ke, Jingping Yuan

**Affiliations:** Department of Pathology, Renmin Hospital of Wuhan University, Wuhan, China

**Keywords:** clear cell variant type, clinicopathologic features, differential diagnosis, molecular features, papillary thyroid carcinoma

## Abstract

The clear cell variant of papillary thyroid carcinoma (CLCVPTC) is an exceedingly rare and diagnostically challenging subtype of papillary thyroid carcinoma (PTC), defined by distinctive histomorphologic features. Here, we aimed to delineate the clinicopathologic, immunohistochemical, and molecular genetic characteristics of CLCVPTC by retrospectively analyzing four pathologically confirmed cases diagnosed at our institution between 2018 and 2025, together with a comprehensive review of the published literature. The male to female ratio of the four CLCVPTC cases is 1: 3, with a median age of 60 years (range 45-68) and presented clinically with thyroid nodules. Histopathological evaluation demonstrated infiltrative tumor growth patterns with solid, trabecular, and focal papillary architectures. Tumor cells displayed abundant clear cytoplasm (more than 90% of all tumor cells in our cases, meeting the diagnostic threshold of >50%) and classic nuclear features of PTC, including ground-glass nuclei, nuclear grooves, and intranuclear inclusions. Lymph node metastases were observed in three cases. Immunohistochemical profiling revealed consistent positivity for TTF-1, Pax8, CK7, thyroglobulin (TG) and Galectin-3, and absence of expression for TPO, BRAF V600E mutation, and various neuroendocrine markers. Polymerase chain reaction (PCR) identified an *NCOA4-RET* gene fusion in 50% (2/4) of the cases. All patients underwent thyroidectomy and central lymph node dissection, among which three of them also underwent contralateral thyroidectomy, with no evidence of disease recurrence during follow-up periods ranging from 2 to 54 months. However, due to the short follow-up for some cases and the loss to follow-up, long-term outcomes for CLCVPTC remain undefined. In conclusion, CLCVPTC is a rare variant of PTC characterized by distinctive clear-cell change with canonical PTC nuclear features. The detection of an *NCOA4-RET* fusion in half of our cases suggests a recurrent genetic alteration that may contribute to its pathogenesis, though this finding requires validation in larger cohorts.

## Background

Papillary thyroid carcinoma (PTC) is the most common thyroid malignancy, accounting for more than 85% of all thyroid cancers (1). CLCVPTC is a rare subtype of PTC and poses a diagnostic challenge because of its distinctive histologic features and extremely low incidence (<1% of thyroid cancer cases) (2). Histologically, CLCVPTC is characterized by prominent clear-cell change, often accompanied by cytoplasmic hyalinization, together with the canonical nuclear features of PTC; consequently, it can be mistaken for other thyroid carcinoma subtypes or for metastatic clear cell carcinomas involving the thyroid (3). Because its clinical and ultrasonographic appearances-such as lesion margins and echogenicity-substantially overlap with those of conventional PTC, accurate preoperative differentiation is difficult. In this study, we describe the clinicopathologic and molecular characteristics of four cases of CLCVPTC and review the relevant literature to refine key differential diagnostic considerations and discuss clinical management, with the goal of improving recognition of this entity and informing more precise patient care.

## Materials and methods

### Clinical information and diagnosis

From January 2018 to December 2025, the Department of Pathology at Renmin Hospital of Wuhan University diagnosed a total of 18,431 cases of PTC, among which 4 were confirmed as CLCVPTC, accounting for approximately 0.02% of all PTC cases during the same period. The diagnosis of these cases was established in accordance with the criteria outlined in the WHO Classification of Tumors, 5th Edition. Specifically, the diagnosis of CLCVPTC required: (1) classic PTC nuclear features (ground-glass nuclei, nuclear grooves, and intranuclear pseudoinclusions); and (2) clear cell change comprising >50% of the tumor cell population, in accordance with previously published criteria (4). All four cases in this series exhibited clear cell change in >90% of tumor cells, substantially exceeding the minimum diagnostic threshold. During the study period, no cases with clear cell change between 50-90% were encountered; therefore, no cases were excluded based on the proportion of clear cells. The specific diagnostic workflow is illustrated in **Supplementary Figure 1**. The study cohort comprised a total of four patients. The group included one male and three female patients. The median age was 60 years, with an age range of 45 to 68 years. The four cases were closely followed up and observed from the date of diagnosis, and the follow-up date was up to November 15, 2025. All pathological sections were reviewed and confirmed by two independent pathologists.

### Methods

Surgically excised specimens were fixed in 4% neutral buffered formaldehyde, followed by conventional dehydration, paraffin embedding, and preparation of 5μm sections. The sections were subsequently stained with hematoxylin and eosin(H&E) and immunohistochemical staining using the EnVision two-step method. The primary antibodies employed included TTF1, Pax8, CK7, CK19, Galectin-3, calcitonin (CT), thyroid peroxidase (TPO), carcinoembryonic antigen (CEA), CD10, CD56, synaptophysin (SYN), chromogranin A (CgA), S100 and p53. The immunohistochemistry kits were procured from Dako, while the BRAF V600E antibody and its corresponding kit were obtained from Roche. The fusion gene was detected using the Amplification Refractory Mutation System PCR (ARMS-PCR), performed with the Aide Bio combined thyroid 10-gene assay kit (Cat. No.240904C02Z). This kit is designed to detect mutations in genes including *BRAF, NRAS, HRAS, KRAS, TERT, PIK3CA, RET, NTRK1, NTRK3*, and *PPARG*. The assay does not detect other *RET* fusion partners and cannot characterize precise fusion breakpoints. The primer sequences employed in this commercial kit constitute proprietary intellectual property of the manufacturer and, as such, are not authorized for public disclosure. The complete product specification sheet, which details the assay’s validation parameters including its reported clinical sensitivity and specificity, is available for review upon request.

## Results

### Clinical features

The clinical data of the four CLCVPTC cases are summarized in **Table 1**. The male-to-female ratio for Cases 1 through 4 is 1: 3, with a median age of 60 years (range 45-68). Cases 1 and 4 were admitted to the hospital due to an anterior cervical mass for more than a month and one year, while Cases 2 and 3 had thyroid goiters detected during physical examinations. Ultrasonographic findings for Cases 1 and 2 revealed mixed masses with calcifications in the right lobe of the thyroid gland (TI-RADS grade 4a), a hypoechoic nodule with calcifications (TI-RADS grade 4C) for Case 4, whereas ultrasonography for Case 3 suggested a thyroid mass (TI-RADS grade 3). Ultrasound of the neck lymph nodes in four cases all suggested lymph node enlargement. Laboratory tests for thyroid function, including T3, T4, and TSH levels, were within normal limits for all four cases. Apart from Case 2, which underwent unilateral thyroidectomy in conjunction with central lymph node dissection, all the other patients received bilateral thyroidectomy accompanied by central lymph node dissection. Postoperative pathology confirmed multiple lymph node metastases in Cases 1, Case 2 and 4, while no lymph node metastases were observed in Case 3. Additionally, Case 1 exhibited Hashimoto’s thyroiditis, and Case 3 and 4 demonstrated papillary carcinoma and CLCVPTC in the contralateral thyroid lobe. During postoperative follow-up, Case 1 was lost to follow-up, whereas Case 2, 3 and 4 were followed up for 54 months, 9 months and 2 months, respectively. Four of them received levothyroxine therapy, radioiodine treatment for Case 4 and neither case exhibited recurrence or distant metastasis during the follow-up period. It is important to note that the follow-up duration for all cases is limited.

**Table 1:** Clinical data of the four cases of CLCVPTC.

Case	Age/Sex	Clinical symptoms	Tumor Size (cm)	TI-RADS	Surgery	Concomitant diseases	LNM	*RET* Fusion status	Follow-up
1	64/Female	Anterior cervical mass for more than a month	2.2	4a	Bilateral + CLND	Hashimoto’s thyroiditis in the other thyroid gland, breast fibroadenoma in the breast	Yes (multiple)	Positive (*NCOA4-RET*)	Lost
2	54/Female	Physical examination revealed a thyroid mass for more than 2 weeks	2.3	4a	Unilateral + CLND	None	Yes (multiple)	Negative	54months without recurrence
3	53/Female	Physical examination revealed a thyroid mass for more than 1 month	0.3	3	Bilateral + CLND	The other side of the thyroid is a classic papillary thyroid carcinoma.	No	Negative	9months without recurrence
4	70/Male	Thyroid noduleswere discovered over a year ago	2.6	4c	Bilateral + CLND	Both sides of the thyroid are CLCVPTC.	Yes (multiple)	Positive *(NCOA4-RET*)	2months without recurrence

CLND, central lymph node dissection; LNM, lymph node metastasis.

### Pathological features

On gross examination, the masses were ill-defined relative to the surrounding thyroid parenchyma. The cut surfaces were gray-white and firm, with focal calcifications. The maximum tumor diameters in the four cases were 2.2 cm, 2.3 cm, 0.3 cm, and 2.6 cm, respectively.

**Figure 1:**
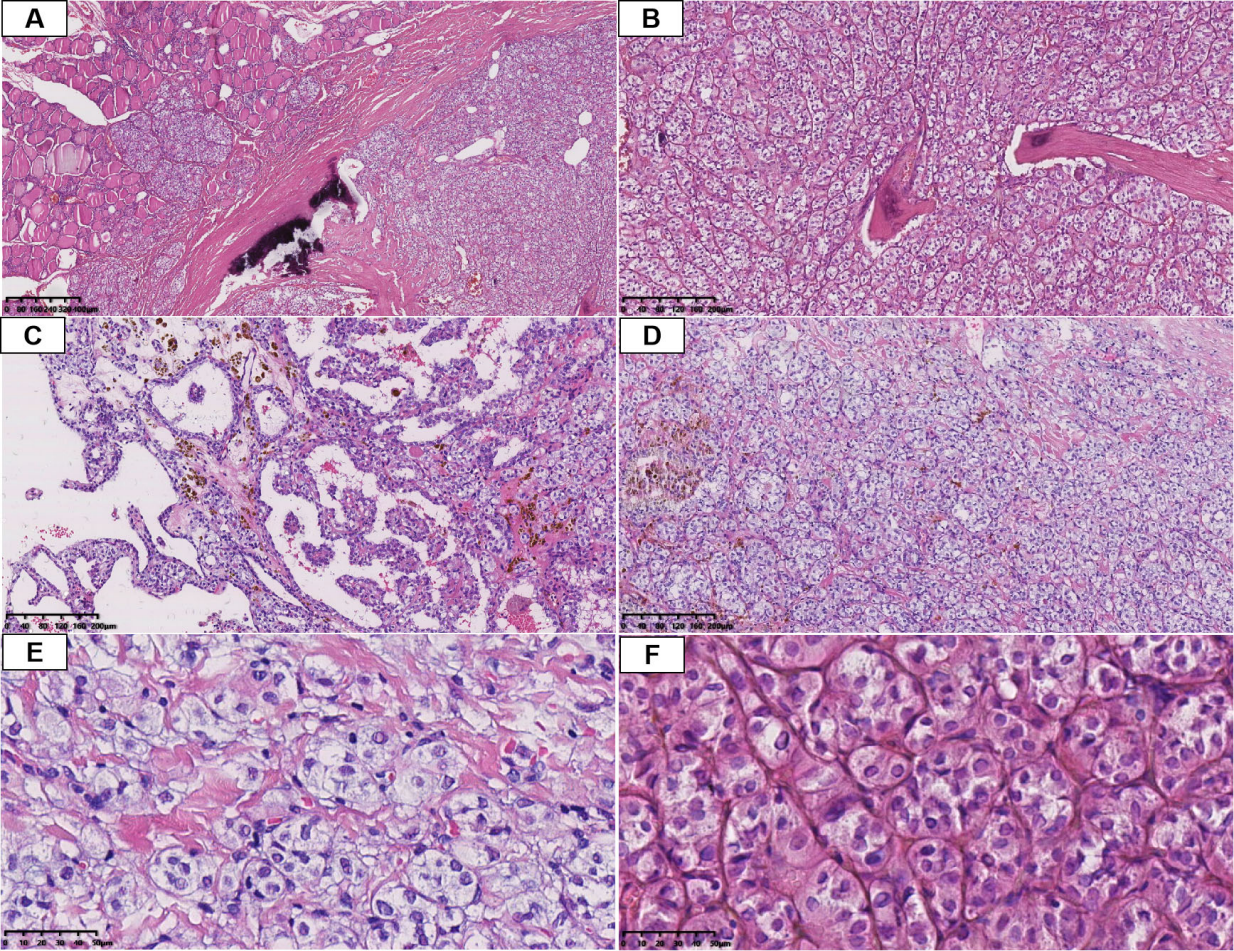
Histopathological features of CLCVPTC. **(A)** Tumor cells infiltrating adjacent normal thyroid tissue with calcifications (H&E, 40×). **(B)** Solid nests of tumor cells with prominent interstitial fibrosis (H&E, 100×). **(C)** Focal papillary architecture (H&E, 100×). **(D)** Hemosiderin deposition and fibrous stromal hyperplasia (H&E, 100×). **(E)** Polygonal tumor cells with abundant clear cytoplasm (H&E, 400×). **(F)** Classic PTC nuclear features including nuclear grooves and intranuclear pseudoinclusions (H&E, 400×).

At low power, the tumors infiltrated adjacent thyroid tissue and were composed predominantly of follicular structures and sheet-like/solid areas (**Figure 1A**). The neoplastic cells formed solid nests and trabeculae within a stroma showing prominent fibrosis and calcification (**Figure 1B**). Focal papillary architecture was also identified (**Figure 1C**). In some areas, the tumor showed stromal expansion with hyalinized change and abundant coarse cytoplasmic granular pigment (**Figure 1D**). Clear cells accounted for >90% of the tumor cell population in all cases, fulfilling the diagnostic criteria for CLCVPTC, which typically requires that clear cells constitute more than 50% of the tumor cells. On high power, tumor cells were relatively uniform, polygonal to round, with abundant clear to pale eosinophilic cytoplasm, sometimes with hyaline change. The nuclei exhibited the classic nuclear features of PTC, including optically clear “ground-glass” nuclei, nuclear grooves, and intranuclear pseudoinclusions (**Figures 1E, F**).

**Figure 2:**
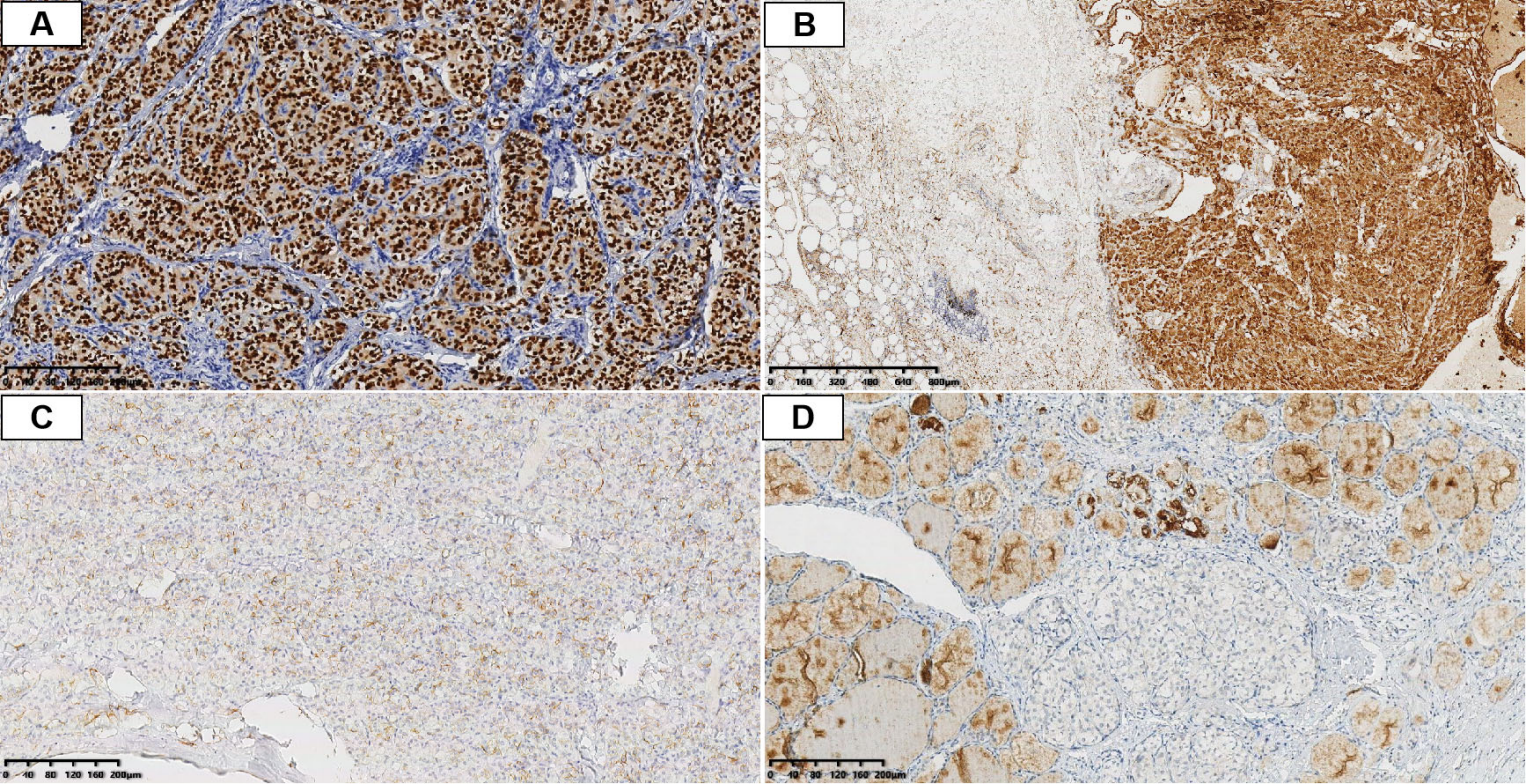
Immunohistochemical profile of CLCVPTC. **(A)** Diffuse nuclear TTF-1 positivity (IHC, 100×). **(B)** Strong cytoplasmic Galectin-3 positivity in tumor cells with negative staining in adjacent normal follicles (IHC, 100×). **(C)** Negative TPO expression in tumor cells (IHC, 100×). **(D)** Negative BRAF V600E staining in tumor cells (IHC, 100×).

Immunohistochemical staining showed diffuse and strong nuclear positivity for TTF-1 (**Figure 2A**) and PAX8, with Galectin-3 positivity (**Figure 2B**). CK7 and CK19 were diffusely positive. TPO was negative (**Figure 2C**). Calcitonin, CEA, CD10, CD56, synaptophysin, chromogranin A, S-100, and BRAF V600E were negative (**Figure 2D**).

**Figure 3:**
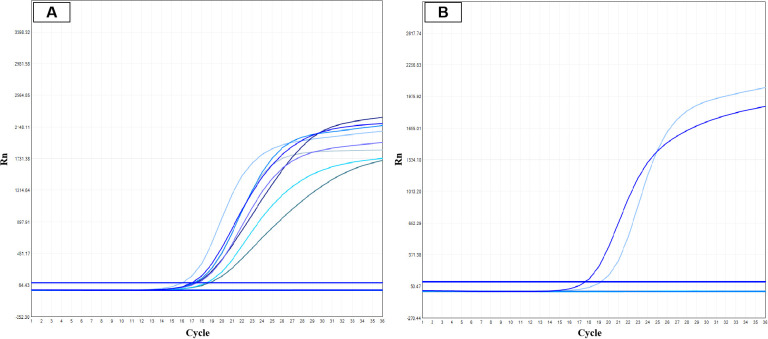
ARMS-PCR detection of *NCOA4-RET* fusion. **(A)** Quality control curves for negative and positive controls. **(B)** *RET* gene fusion curve (dark blue) and internal quality control curve (light blue).

Four cases were tested for 10 common genes of thyroid tumors by ARMS-PCR. Case 1 and 4 showed *RET* gene fusion (**Figure 3**), Cases 2 and 3 were negative.

## Discussion

Papillary thyroid carcinoma (PTC), the most common malignancy arising from thyroid follicular epithelium, is diagnostically defined by its characteristic nuclear features, including ground-glass nuclei, nuclear grooves, and intranuclear pseudoinclusion (5). CLCVPTC is a rare subtype, accounting for <1% of cases, and poses a diagnostic challenge because of its distinctive cytomorphology—most notably cytoplasmic clearing, which may result from glycogen accumulation or organelle swelling—as well as its low incidence (6). In PTC, cytoplasmic clearing is often associated with intracellular glycogen accumulation (7).

Clear-cell change may be encountered in a spectrum of thyroid and parathyroid neoplasms and can arise through diverse mechanisms. Because cytoplasmic clearing is not specific to CLCVPTC, a broad differential diagnosis is required. Foremost, metastatic clear cell tumors involving the thyroid—particularly clear cell renal cell carcinoma—must be excluded. In this setting, metastatic renal cell carcinoma is typically negative for thyroid lineage markers and positive for markers such as CAIX and RCC. Additional diagnostic considerations include parathyroid neoplasms (TTF-1–negative, PTH-positive), thyroid oncocytic neoplasms, hyalinizing trabecular tumor (often associated with PAX8–GLIS3 fusion), medullary thyroid carcinoma (positive for neuroendocrine markers), and paraganglioma (with S100–positive sustentacular cells) (8–14). Accurate diagnosis therefore relies on a comprehensive immunohistochemical panel interpreted in conjunction with careful clinical correlation. The key differential diagnoses of CLCVPTC, along with their distinguishing morphologic, immunophenotypic, and molecular features, are summarized in **Table 2**.

**Table 2:** Differential diagnosis of CLCVPTC.

Tumor type	Morphologic features	Immunophenotype	Molecular features
CLCVPTC	Clear cytoplasm (>50%), classic PTC nuclear features (ground-glass nuclei, grooves, pseudoinclusions), papillary/solid/trabecular growth	TTF-1(+), PAX8(+), CK7(+), TG(+), Galectin-3(+), CK19(+), TPO(-), BRAF V600E(-), neuroendocrine markers(-)	*NCOA4-RET* fusion (in subset), other PTC-related fusions possible
Metastatic RCC	Abundant clear cytoplasm, nested/alveolar growth, prominent vascular network, often lacks PTC nuclear features	PAX8(+), TTF-1(-), TG(-), CK7(–/+), CAIX(+), RCC(+), CD10(+), vimentin(+)	VHL mutations, 3p deletion, clear cell RCC-associated alterations
Parathyroid carcinoma	Chief cells with clear or eosinophilic cytoplasm, trabecular growth, fibrous bands, capsular/vascular invasion	PTH(+), GATA3(+), TTF-1(-), PAX8(-) (or weak), CK7(-), chromogranin(+) (variable)	CDC73 mutations, loss of parafibromin expression
Hyalinizing trabecular tumor	Trabecular growth with hyalinized stroma, spindle-shaped cells, nuclear grooves, cytoplasmic yellow bodies	TTF-1(+), PAX8(+), TG(+), membranous MIB-1 (unique), *RET*(-), neuroendocrine markers(-)	PAX8-GLIS3 fusion, RAS mutations rare
Medullary thyroid carcinoma	Plasmacytoid/spindle cells, neuroendocrine chromatin, amyloid stroma (common but not invariable)	CT(+), CEA(+), chromogranin(+), synaptophysin(+), TTF-1(+), PAX8(+), TG(-)	*RET* mutations (germline/somatic), RAS mutations

In addition to histopathological and immunohistochemical distinction, ultrasonographic features may provide complementary information in the differential diagnosis of PTC subtypes. Hekimsoy et al. (15) analyzed 142 PTC nodules based on the 2022 WHO classification and found that classic PTC and tall cell subtype more frequently demonstrated malignant ultrasound features such as irregular margins, echogenic foci, and higher TIRADS categories, whereas IEFV-PTC and NIFTP rarely exhibited these high-risk features. We observed that the four CLCVPTC cases presented with TI-RADS grades ranging from 3 to 4c, with features including hypoechoic or mixed echogenicity, irregular margins, and calcifications. This ultrasound phenotype appears to align more closely with that of classic PTC and tall cell subtype than with indolent variants, which is consistent with the observed nodal metastasis in three of our cases.

In this study, we present four cases of CLCVPTC where the tumor cells exhibited both classic PTC nuclear features and over 50% of cytoplasmic clear cells, aligning with the core pathological diagnostic criteria for this subtype (4, 16). Furthermore, immunohistochemical analysis revealed diffuse positivity for Galectin-3 and CK19 in all cases, consistent with the molecular profile of conventional PTC and aiding its distinction from benign thyroid lesions.

A significant finding of our study was the detection of *NCOA4-RET* gene fusions in two of the four cases. According to the 5th edition WHO classification, PTCs harboring fusion genes such as *RET* or *NTRK* are often categorized as RAS-like tumors, which typically demonstrate infiltrative growth patterns and characteristic PTC nuclear morphology. In our series, both *RET* fusion-positive cases (Cases 1 and 4) exhibited lymph node metastases. However, lymph node metastasis was also observed in one *RET* fusion-negative case (Case 2), indicating that while *RET* fusion may contribute to aggressive behavior in some tumors, it is not the sole determinant of metastatic potential. Given the small sample size and the presence of *RET*-negative cases with nodal involvement, these findings should be considered hypothesis-generating and require validation in larger cohorts. A detailed comparison of clinicopathologic features by *RET* status is presented in **Table 1**. Notably, this is a preliminary observation that requires confirmation in larger, multi-institutional cohorts using orthogonal methods such as FISH or NGS.

*RET* fusions are well-established driver events in conventional PTC, particularly in radiation-associated and pediatric cases. However, their prevalence and significance in CLCVPTC have not been systematically evaluated. A review of the literature reveals that molecular characterization of CLCVPTC is extremely limited, with most published cases lacking comprehensive genetic analysis. To our knowledge, this is the first case series to report the presence of *NCOA4-RET* fusions in a subset of CLCVPTC. While *RET* fusions are not specific to this variant, as they are known to occur in a subset of conventional PTC, particularly those arising in children or following radiation exposure (17, 18), their identification in 50% of our cases suggests that this alteration may be a recurrent finding in CLCVPTC, warranting further investigation in larger cohorts.

Regarding management, total thyroidectomy with central compartment lymph node dissection has been the most commonly reported surgical approach for CLCVPTC (19–22). In our series, the presence of lymph node metastases suggests that this variant may have a propensity for nodal involvement. Accordingly, even in clinically node-negative (cN0) disease, a lower threshold for considering prophylactic central neck dissection may be reasonable to optimize pathologic staging and inform postoperative risk stratification and adjuvant management. From a translational standpoint, identification of a *RET* fusion has important therapeutic implications. For the minority of patients who develop refractory, recurrent, or metastatic disease, selective *RET* tyrosine kinase inhibitors may offer an effective targeted treatment option.

In conclusion, our analysis of four cases further delineates the clinicopathologic spectrum of CLCVPTC and provides preliminary evidence that *RET* gene fusions may contribute to its molecular pathogenesis. It is important to acknowledge that the small sample size, particularly the identification of *RET* fusion in only two cases, is a key limitation of this study and precludes definitive statistical conclusions or claims regarding its role as a molecular hallmark. Furthermore, the follow-up data in this study are incomplete and of limited duration for some patients (as short as 2 months), and one patient was lost to follow-up. Given the extreme rarity of this variant, standardized diagnostic and management guidelines are lacking, and its long-term prognosis and optimal treatment strategies remain to be defined through larger case series and more detailed studies. Improving recognition of this entity, applying key diagnostic criteria, and exploring individualized therapeutic approaches will be important for optimizing patient outcomes.

## Conclusion

CLCVPTC is a rare variant of PTC characterized by prominent clear cell change in conjunction with classic PTC nuclear features, necessitating distinction from metastatic clear cell tumors and other thyroid neoplasms. The detection of *NCOA4-RET* fusions in half of our cases suggests a recurrent genetic alteration in this variant, though this observation requires validation in larger cohorts. Due to the small sample size and limited follow-up, the long-term prognosis of CLCVPTC remains uncertain.

## Data Availability

The raw data supporting the conclusions of this article will be made available by the authors, without undue reservation.

## Supplementary material

Supplementary Figure 1 The specific diagnostic workflow of CLCVPTC.
