# Supplementary figures and images for: Case Report: The clear cell variant of papillary thyroid carcinoma: a clinicopathologic study of four cases with emphasis on RET gene fusions
Source: Front Oncol. 2026 Apr 23;16:1787485. doi: 10.3389/fonc.2026.1787485 (PMC13149142; doi:10.3389/fonc.2026.1787485)

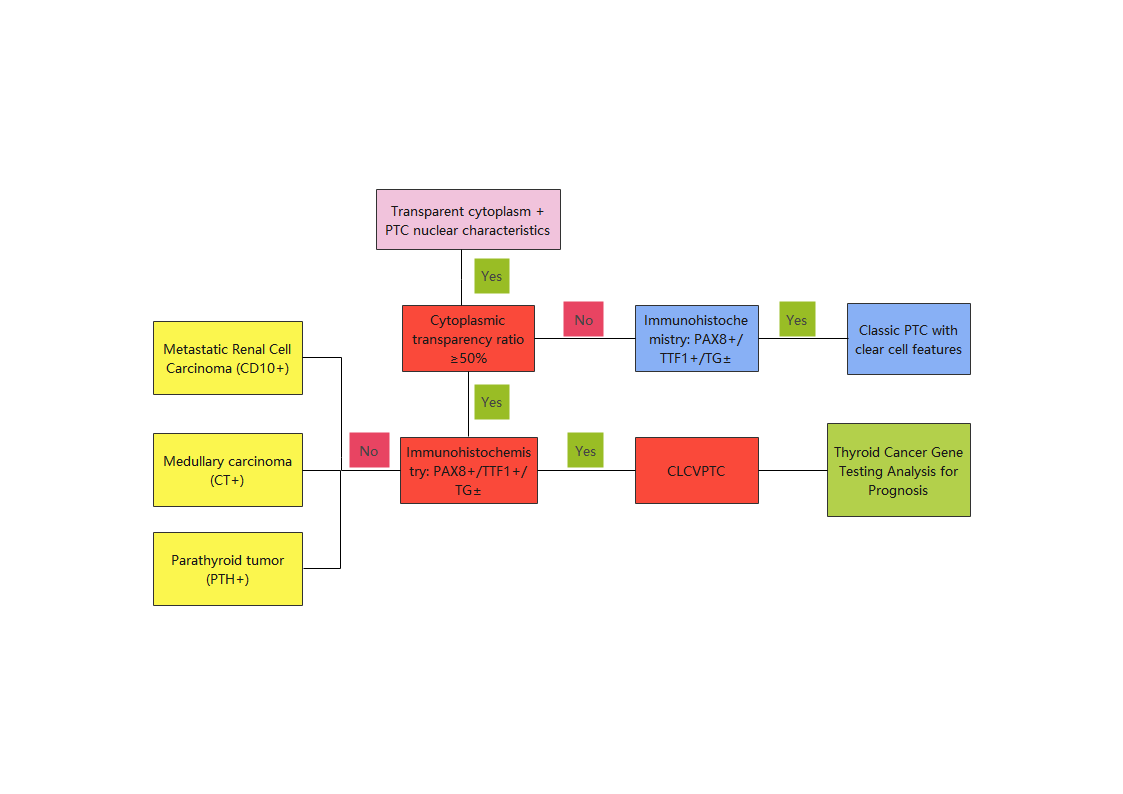

Supplement: Supplementary Figure 1 — The specific diagnostic workflow of CLCVPTC. [file Image1.tiff]
